# Macrophage signaling associates with fibrogenic program activation in periductal fibroblasts in pediatric primary sclerosing cholangitis

**DOI:** 10.1172/jci.insight.199226

**Published:** 2026-04-23

**Authors:** Yunguan Wang, David Adeleke, Xiangfei Xie, Zi F. Yang, Xiangya Wang, Giulia Loi, Annika Yang vom Hofe, Manavi Singh, Astha Malik, Ramesh Kudira, Cyd Castro-Rojas, Liva Pfuhler, Mosab Alquraish, Pamela Sylvestre, Jonathan R. Dillman, Andrew T. Trout, Emily R. Miraldi, Alexander G. Miethke

**Affiliations:** 1 Division of Pediatric Gastroenterology, Hepatology and Nutrition,; 2 Division of Human Genetics, and; 3 Divisions of Immunobiology and Biomedical Informatics, Cincinnati Children’s Hospital Medical Center, Cincinnati, Ohio, USA.; 4 Department of Pediatrics, University of Cincinnati College of Medicine, Cincinnati, Ohio, USA.; 5 Molecular Developmental Biology Graduate Program, Cincinnati Children’s Hospital Medical Center, Cincinnati, Ohio, USA.; 6 Department of Mathematical Sciences, University of Cincinnati, Cincinnati, Ohio, USA.; 7 Biomedical Informatics Graduate Program and; 8 Division of Pathology and Laboratory Medicine, Cincinnati Children’s Hospital Medical Center, Cincinnati, Ohio, USA.; 9 Department of Pathology and Laboratory Medicine, University of Cincinnati College of Medicine, Cincinnati, Ohio, USA.; 10 Department of Radiology, Cincinnati Children’s Hospital Medical Center and University of Cincinnati College of Medicine, Cincinnati, Ohio, USA.; 11 Divisions of Immunobiology and Biomedical Informatics, Cincinnati Children’s Hospital Medical Center, Cincinnati, Ohio, USA.

**Keywords:** Autoimmunity, Gastroenterology, Bioinformatics, Fibrosis, Macrophages

## Abstract

Primary sclerosing cholangitis (PSC) is a chronic, idiopathic cholestatic liver disease characterized by inflammation and fibrosis of the bile ducts, yet the cellular crosstalk driving periductal fibrosis remains poorly defined. This study applied a multiomics approach integrating spatial transcriptomics, RNA-Seq, and proteomics to characterize fibrotic periductal regions and their cell-cell communications. Macrophage subsets, including monocyte-derived macrophages and lipid-associated macrophage–like cells, colocalized with cholangiocytes, lymphocytes, and hepatic stellate cells (HSCs). Cell niche analysis identified periductal regions with elevated fibrotic signals, where cell-cell communication analysis revealed potential macrophage-HSC interactions involving 17 fibrotic driver genes in macrophages, including *ITGB2*, *GRN,* and *CCL21*, and 6 fibrotic effector genes in HSCs. In validation analyses, bulk RNA-Seq data showed higher driver and effector gene expression in PSC with established fibrosis compared with early-stage PSC or healthy controls. Plasma proteins encoded by macrophage driver genes were elevated in PSC and in patients with elevated (≥3.29 kPa) liver stiffness on MR elastography. Immunofluorescence and second harmonic generation imaging showed enrichment of CD68^+^/CD18^+^(*ITGB2*) macrophages in fibrotic regions of PSC liver biopsies. These findings revealed enrichment of monocyte-derived macrophages and lipid-associated macrophage–like cells in fibrotic regions and suggest that they likely contribute to fibrotic activation of nearby HSCs in PSC.

## Abstract 2

This study identifies immune cells that gather around bile ducts in PSC and may activate nearby cells that drive liver scarring.

## Introduction

Primary sclerosing cholangitis (PSC) is a chronic, idiopathic cholestatic liver disease characterized by inflammation and fibrosis of the bile ducts. Although the exact etiology of PSC remains unclear, GWAS have implicated genes highly expressed in lymphocytes and myeloid cells — such as HLA genes, FCRL3, INAVA, PRDM1, CCR6, CD226, and IL12RB1 — as risk factors for developing PSC (1). PSC is a slowly but relentlessly progressive disease in children and adolescents, with 38% and 25% developing portal hypertensive and biliary complications, respectively, within 10 years of disease onset and 14% of patients requiring liver transplantation to prolong survival, as shown in a large, multicenter, retrospective cohort study (2). Unique to children and adolescents is the overlap with autoimmune hepatitis (AIH) in 33% of patients with PSC, whereas patients of all ages are frequently diagnosed with concomitant inflammatory bowel disease (IBD). Current treatments focus on managing symptoms and complications, but no approved therapy effectively halts hepatobiliary fibrosis progression (3).

The development of hepatobiliary fibrosis involves complex interactions between several cell types, including cholangiocytes, fibroblasts (portal fibroblasts and hepatic stellate cells [HSCs]), and immune cells. In mouse models of sclerosing cholangitis, injury to bile duct epithelial cells activates nearby fibroblasts, which then migrate to bile ducts, accelerating liver fibrosis progression (4–7). Both portal fibroblasts and recruited HSCs contribute to biliary fibrosis by adopting a myofibroblast phenotype characterized by ACTA2 upregulation and excessive collagen production in mouse models of PSC (8).

Macrophages also play a critical role in PSC pathogenesis. These cells, known for their diverse functions, have both proinflammatory and fibrogenic properties (6). Macrophages promote inflammation and liver damage by secreting cytokines that recruit and activate other immune cells. Additionally, macrophages contribute directly to fibrosis by activating HSCs and producing fibrogenic cytokines such as TGF-β (5). Although macrophages have been identified as key drivers of hepatobiliary fibrosis in PSC, their precise roles across macrophage subpopulations remain poorly understood. Guicciardi et al. demonstrated that inhibiting macrophage recruitment reduced peribiliary fibro-inflammation in an Mdr2^–/–^ mouse model of sclerosing cholangitis (5). In contrast, in a cholestatic injury model involving bile duct ligation and dietary 3,5-diethoxycarbonyl-1,4-dihydrocollidine, macrophage depletion during early cholestasis induction had no significant impact on fibrosis progression (9, 10).

Recent studies investigating PSC liver immunology in humans revealed that macrophages exist in distinct subtypes, including Kupffer cells, monocyte-like macrophages (MoMPs), and lipid-associated macrophage–like (LAM-like) cells (10, 11). Spatially resolved transcriptomics (SRT) analysis using the Visium platform showed that these macrophage subtypes are distributed differently across the portal-central axis and between scarring and non-scarring regions. However, the limited resolution of the Visium platform leaves gaps in our understanding of how macrophages are spatially organized within fibrotic niches and how their transcriptomic heterogeneity contributes to local fibrotic gene programs.

**Figure 1:**
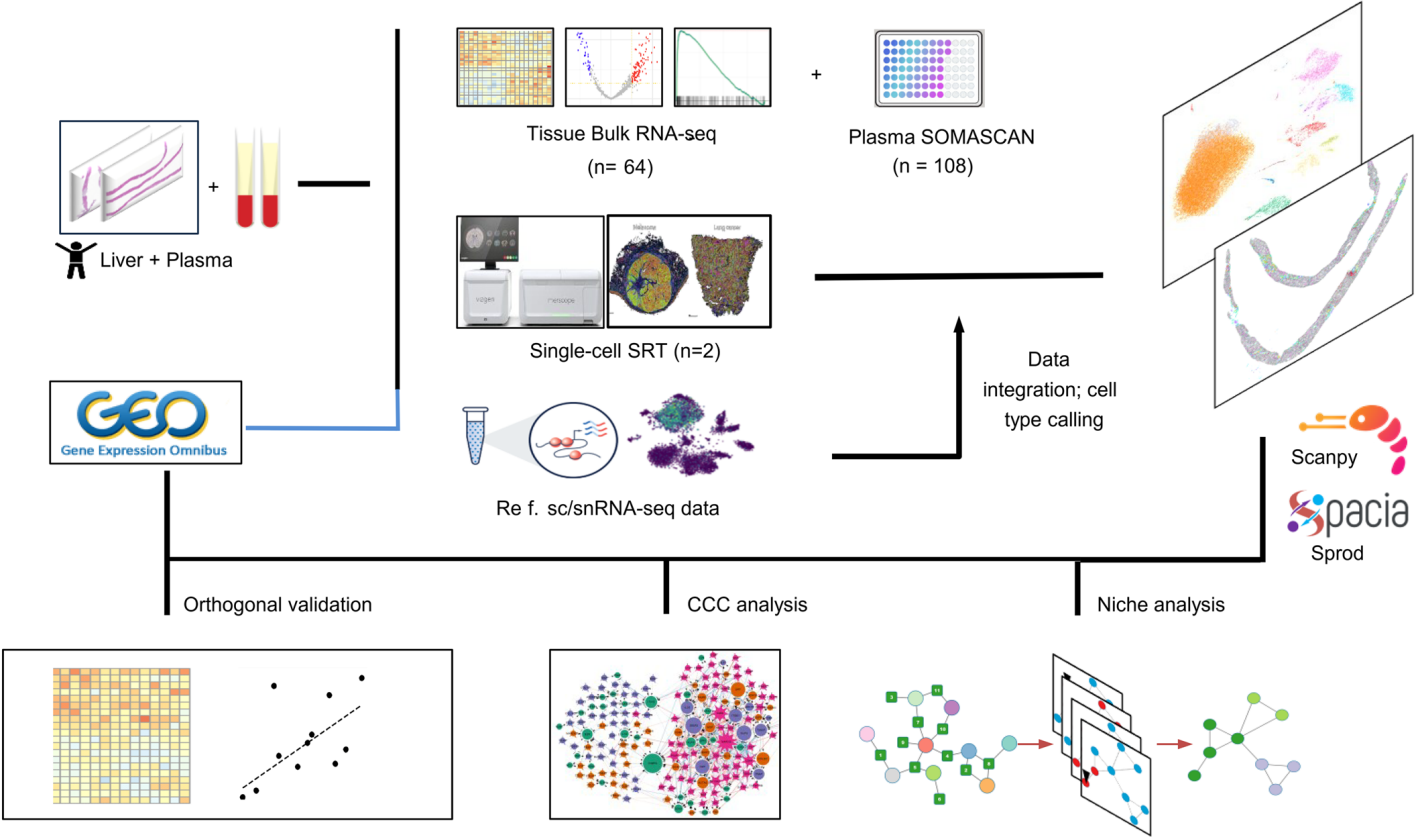
Analytical workflow for scSRT data analysis, integration, CCC prediction, and orthogonal validation.

To address these gaps, we employed a multiomics approach integrating single-cell spatially resolved transcriptomics (scSRT), RNA-Seq, and plasma proteomics data from patients with autoimmune liver disease (AILD) and healthy donors (*n* = 2, 64, and 108, respectively) across the different platforms to investigate the role of macrophages in fibroblast activation (the workflow is summarized in Figure 1). Using unsupervised cell niche analysis, we identified fibrotic regions in liver biopsies and characterized the macrophage populations enriched within them. We then prioritized key cell-cell communication (CCC) genes that may mediate interactions between macrophages and fibrogenic fibroblasts. We hypothesized that gene programs identified through CCC in scSRT data would also exhibit strong associations in non-spatial data. Consistent with this hypothesis, we confirmed that fibrogenic gene expression in fibroblasts correlates with CCC-associated driver genes in macrophages in PSC scRNA-Seq data and that these fibrotic gene signatures are linked to advanced fibrosis stages in pediatric PSC.

## Results

### Macrophage activity is correlated with advanced fibrosis in AILD.

**Figure 2:**
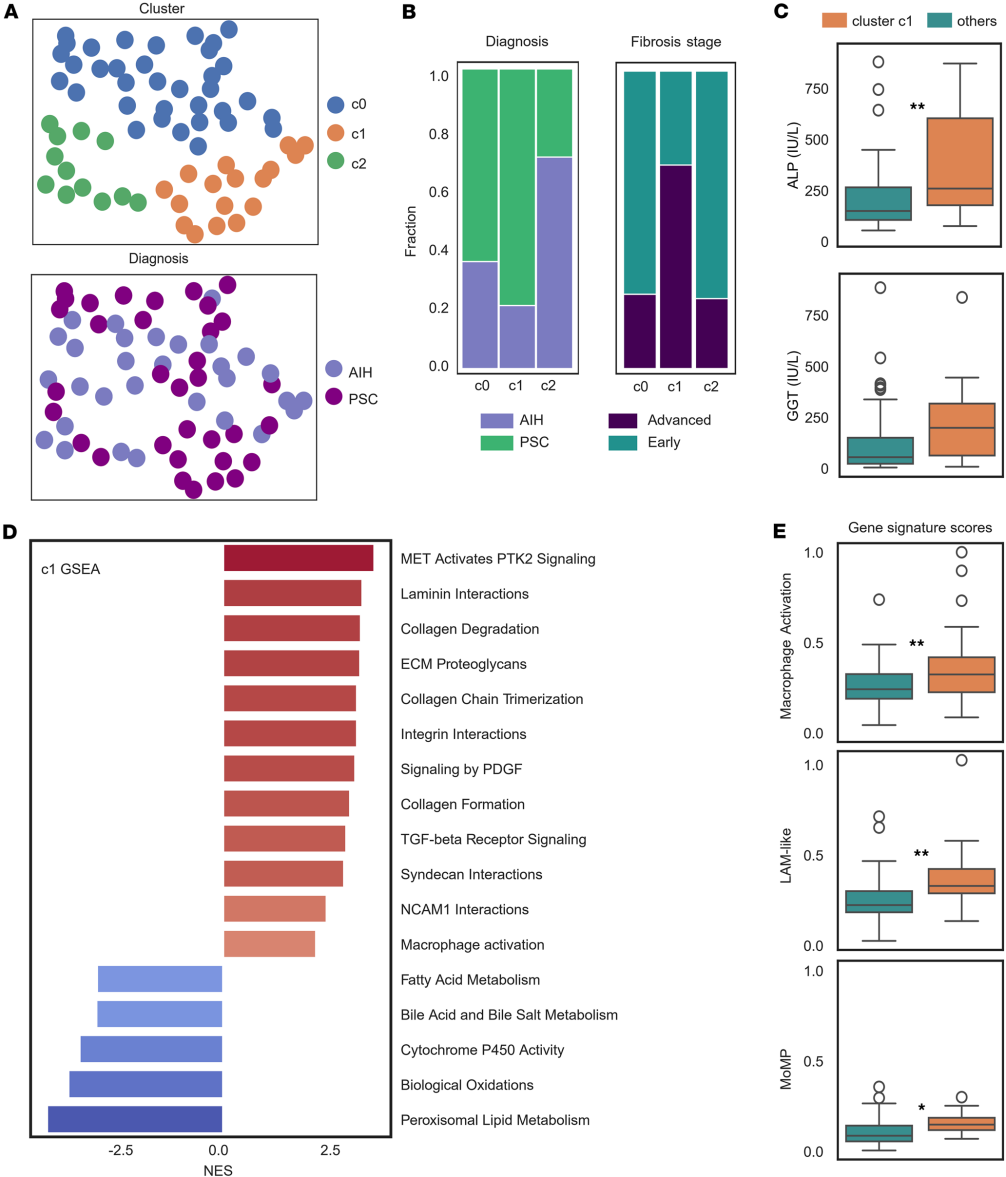
Evaluation of the association between macrophage gene programs and liver fibrosis in PSC. (**A**) Unsupervised clustering 64 AILD patient samples shown in embeddings calculated using UMAP. Samples are colored based on clusters (upper) or patient diagnosis at the time of data collection (lower). (**B**) Compositions of each patient sample cluster, based on diagnosis (left) or fibrosis stage (right). (**C**) Serum GGT and ALP levels in each patient cluster. (**D**) GSEA results calculated from the ranked DEGs in cluster 1 compared with c0 and c2. Pathways shown were selected based on an FDR *q* value cutoff of 0.25 and are colored based on the normalized enrichment score (NES). Both the *q* value and the NES were calculated using the gseapy Python package. (**E**) Average expression of genes involved in macrophage activation and of marker genes for LAM and MoMPs in each patient cluster. Values were normalized to a numeric range of 0–1. For **D** and **E**, significance was obtained from Student’s *t* test comparing c1 versus the other patient clusters: *P < 0.05, **P < 0.01. Whiskers were extended to 1.5 times IQR from Q1 and Q3.

To investigate the association between macrophage activation and fibrosis-associated transcriptional programs across AILD, we performed RNA-Seq of cryopreserved tissue samples from 64 clinically indicated liver biopsies of pediatric patients with PSC or AIH. These samples were collected at the Center for Autoimmune Liver Disease (CALD) at Cincinnati Children’s Hospital Medical Center, and clinical data are summarized in Table 1. Given the potential biases introduced by tissue heterogeneity and bulk RNA-Seq data, we applied unsupervised clustering to identify common proinflammatory and fibrogenic gene expression patterns. This analysis revealed 3 distinct clusters (Figure 2A): cluster c1 was predominantly composed of PSC samples compared with the other 2 clusters (78.4% vs. 51.1% with PSC, OR = 3.587, *P* = 0.052, Figure 2B). Notably, cluster c1 was significantly enriched in patients with advanced fibrosis (OR = 6.697, *P* = 0.002), as defined by a METAVIR score of 2 or higher or an ISHAK score of 3 or higher (Figure 2B). Compared with the rest, patients in c1 exhibited bile duct injuries, as evidenced by higher alkaline phosphatase (ALP) (*P* = 0.004) and serum **γ**-glutamyl transferase (GGT) levels (*P* = 0.07) (Figure 2C). To further explore the relationship between gene expression and fibrosis, we performed Gene Set Enrichment Analysis (GSEA) using genes differentially expressed in cluster c1. We found upregulation of pathways involved in macrophage activation, TGF-β signaling, and collagen formation; genes related to bile and lipid metabolism were among the most downregulated in patients from c1 compared with patients in c0 or c2 (Figure 2D). To further assess macrophage activity in these AILD samples, we calculated the average expression of macrophage-related genes identified in a reference single-cell RNA-Seq (scRNA-Seq) dataset of patients with PSC (10). Genes associated with macrophage activation — including those highly expressed in LAM and MoMP subpopulations — were significantly upregulated in the PSC-dominant cluster c1 (Figure 2E). Genes associated with abundance of LAM included *SPP1, FABP5, CSTB, CD9,* and *GPNMB,* whereas *VCAN, S100A6, S100A4, LYZ, S100A9, S100A12,* and *S100A8* identified MoMP. Collectively, these results suggest that macrophage activity is elevated in patients with AILD and advanced fibrosis, potentially contributing to the activation of fibrotic gene programs.

### Cell types identified by scSRT recapitulate the cellular landscape of PSC.

**Figure 3:**
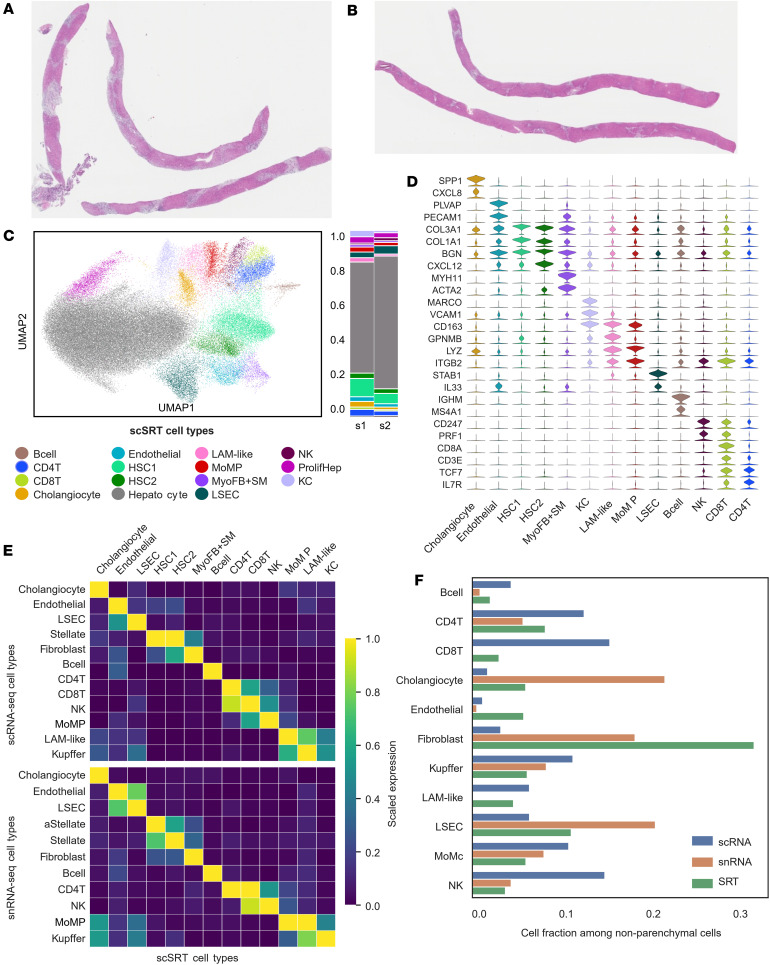
Evaluation of the cell types identified in scSRT PSC samples. (**A** and **B**) Overview of the 2 PSC samples used in this study. FFPE slides were stained with H&E. Scale bar: 1 mm. (**C**) PSC cell types identified using unsupervised clustering shown in embeddings calculated using UMAP. Samples are colored based on cell types. (**D**) Violin plot showing expression of selected marker genes of each cell type. Each violin is colored based on cell types shown in **C**. (**E**) Average expression of top 5 DEGs from each scSRT cell type (rows) in scRNA-Seq cell types (columns) in reference scRNA-Seq (top) and snRNA-Seq (bottom) datasets. Log1p counts per million (CPM) gene expression values were scaled to a range of 0 to 1. (**F**) Fractions of each cell type among all non-hepatocytes in scSRT and scRNA-Seq data.

To investigate the transcriptomic and spatial heterogeneity of these macrophage populations as putative drivers of fibrosis in PSC, we performed scSRT using the VizGen MERSCOPE platform and a custom 400-gene liver immunology panel (Supplemental Table 1; supplemental material available online with this article; https://doi.org/10.1172/jci.insight.199226DS1). Two fresh-cut, archived FFPE slides from diagnostic liver biopsies of 2 patients with pediatric-onset PSC with areas of focal periductal and bridging fibrosis were analyzed. At the time of the liver biopsy, participant A was a 10-year-old female with a new diagnosis of PSC and possible overlap syndrome with AIH and a past medical history of autoimmune pancreatitis and inflammatory bowel disease. Participant B, a 12-year-old male with a history of elevated liver function tests and abnormal liver imaging, was diagnosed with PSC based on the liver biopsy results (Table 2 and Figure 3, A and B). Quality control of the scSRT data included a series of filtering steps based on metrics such as cell size, read counts, and doublet probability. A total of 93,879 cells were retained, with a median per-cell transcript count of 95. To mitigate dropout effects and improve segmentation, we incorporated data denoising and transcript-based re-segmentation in the preprocessing workflow using Sprod (12) and Baysor (13), respectively.

Next, we applied unsupervised clustering and identified major PSC-associated cell types using reference PSC cell-type marker genes. These included hepatocytes, cholangiocytes, liver sinusoidal endothelial cells, macrophages, T cells, and HSCs (Figure 3C). Notably, 2 HSC subpopulations emerged: HSC1, characterized by high expression of classical fibrosis genes *COL3A1* and *COL1A1*, and HSC2, which predominantly expressed *COL6A1* and *CXCL12*. Similarly, 3 macrophage populations were identified: LAM, MoMP, and Kupffer cells (Figure 3D). Interestingly, all 3 macrophage subsets exhibited moderate to high expression of the noninflammatory macrophage marker *CD163*. CD4^+^ T cells highly expressed naive/central-memory markers including *TCF7* and *IL7R,* and tissue-resident markers including *CD69* and *CXCR4*. This phenotype was consistent with the naive-like T cells previously reported in PSC (14). CD8^+^ T cells exhibited cytotoxic and central-memory phenotypes, with high expression of GZMK, PRF1, *TCF7,* and *CD69* (Supplemental Figure 1). Additional differentially expressed genes (DEGs;1 vs. rest) are summarized and reported in Supplemental Table 2.

To assess the concordance between cell types identified in scSRT and those from scRNA-Seq, we used scRNA-Seq and single-nuclei RNA-Seq (snRNA-Seq) data from 16 healthy individuals and patients with PSC or primary biliary cholangitis (PBC), with cell-type annotations provided by the original authors (10). We computed the uniform manifold approximation and projection (UMAP) of single cells from 8 patients with PSC, including 7 with decompensated cirrhosis, using only the 400 MERSCOPE genes, and found that cell types remained well-separated (Supplemental Figure 2). Next, we evaluated the expression of marker genes from our scSRT cell types in the 2 reference datasets. As shown in Figure 3E, marker genes identified from scSRT cell types, including CD4/8^+^ T cells, B cells, macrophages, hepatocytes, and cholangiocytes, were selectively expressed in the corresponding cell types from the scRNA-Seq/snRNA-Seq datasets. Based on the scRNA-Seq dataset, HSC1 from the scSRT corresponded to activated stellate cells, while HSC2 expressed genes found in quiescent stellate cells. These results supported the robustness of the cell-type annotations. We then compared the composition of cell types, excluding hepatocytes, between the 2 liver transcriptomic datasets from patients with PSC. To facilitate interpretation, we grouped HSCs and myofibroblasts into a general fibroblast population. The scRNA-Seq/snRNA-Seq data contained a higher proportion of lymphoid and myeloid cells, whereas scSRT data were enriched with cholangiocytes, endothelial cells, and fibroblasts (Figure 3F). Notably, these results are consistent with earlier IHC- and Visium-based studies showing that T cells, CD163^+^ macrophages (15), SPP1^+^ macrophages (16), and LAM-like cells are enriched within fibrotic portal regions, which are themselves encircled by layers of SMA^+^ myofibroblasts in adult PSC livers (10).

**Figure 4:**
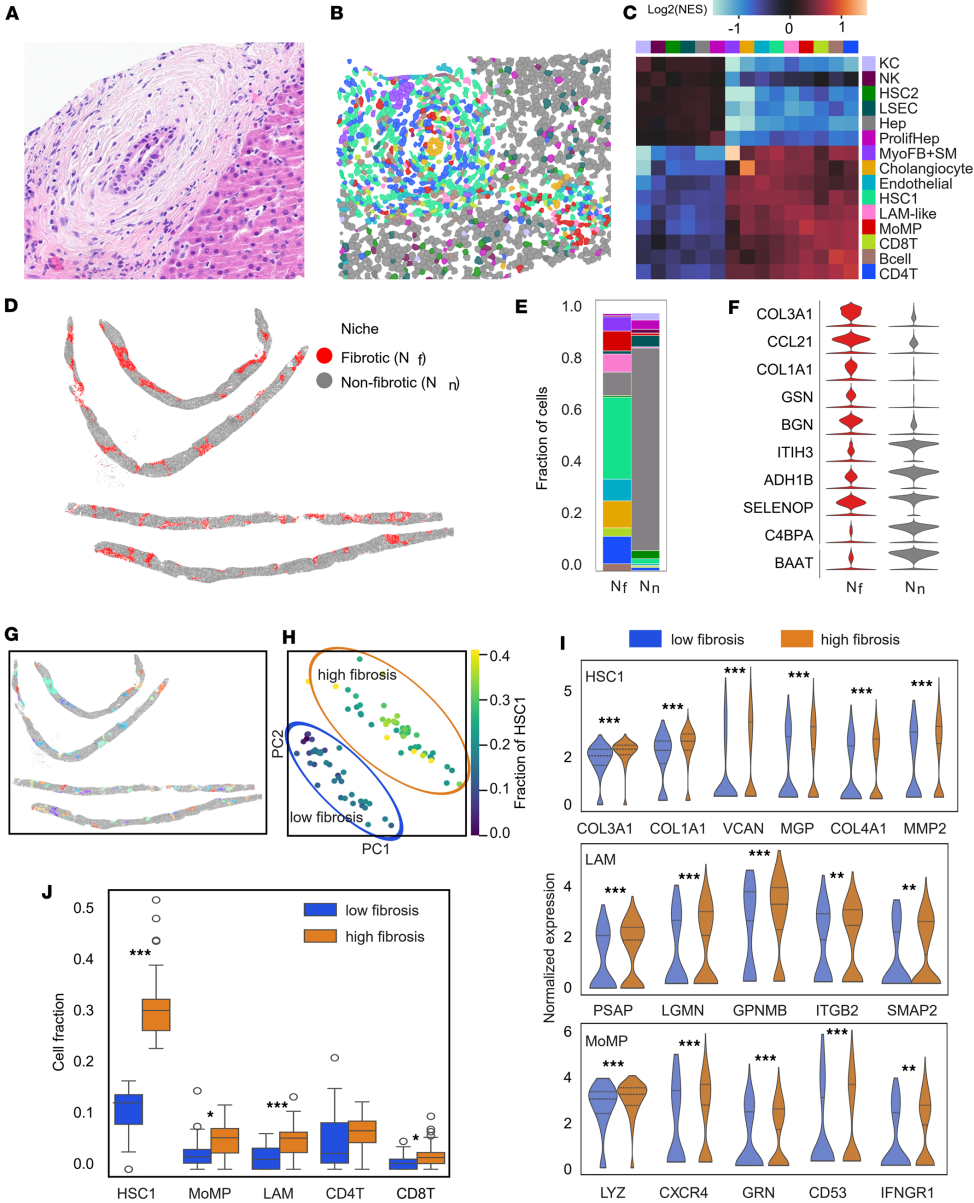
Neighborhood analysis of PSC fibrotic cell niches. (**A**) A region with onion skin fibrosis on a consecutive slice from the same tissue used in scSRT profiling. (**B**) A reconstructed image focusing on a region with onion skin fibrosis using scSRT data. Polygons in the image represent segmented cell masks and are colored based on cell types. (**C**) Heatmap showing neighborhood enrichment scores (NES) between each evaluated neighboring cell type (columns) in the proximity of the reference cell type (rows). Each cell in the heatmap is colored based on the log_2_(NES) value. (**D**) Reconstructed image showing the location of each cell in the scSRT data. Cells are shown as dots on the image and colored based on their niche identity. (**E**) Stacked bar plot showing the fraction of each cell type among all cells in the fibrotic and nonfibrotic niches. (**F**) Top 5 DEGs for the fibrotic and nonfibrotic niches. Differential analysis was performed using Wilcoxon’s rank-sum test with FDR-adjusted *P* value cutoff at 0.05. DEGs were ranked using log fold-changes. (**G**) Reconstructed image of cells in scSRT data showing the location of periductal regions. (**H**) PCA plot calculated from the pseudo-bulk–level expression profiles from periductal regions. Pseudo-bulks are colored based on HSC1 fraction. (**I**) Boxplot showing the enrichment of cell types in the high-fibrosis regions compared with the low-fibrosis regions. Significance obtained from Student’s *t* test across the 2 groups. *P < 0.05, **P < 0.01, ***P < 0.001. (**J**) Violin plot of genes upregulated in the high-fibrosis periductal regions. Differential analysis was performed in each cell type using Wilcoxon’s rank-sum test. DEGs were filtered based on FDR-adjusted *P* value cutoff at 0.05 and minimum log fold-change of 0.6. Quartiles are shown inside each violin.

In regions exhibiting onion-skin fibrosis, a characteristic feature of PSC (Figure 4A), we observed concentric layers of LAM-like cells, MoMPs, HSC1 cells, and T cells surrounding the bile ducts (Figure 4B). To quantitatively assess the differential spatial localization of these cell types, we performed a neighborhood enrichment analysis. We found that HSC1 cells, LAM-like cells, MoMPs, and CD4/8^+^ T cells were significantly enriched around bile duct epithelial cells (at least 168% of expected, *P* < 0.0001), while HSC2 and Kupffer cells were largely excluded from the cholangiocyte neighborhood (HSC2: 34% of expected; Kupffer cells: 15% of expected, *P* < 0.0001, Figure 4C).To further characterize these niches, we used CellCharter (17) and identified 2 spatially distinct cell niches (Figure 4D). The first, termed the fibrotic niche, contained a large fraction of HSC1 cells and was enriched in LAM-like cells, MoMPs, and CD4/8^+^ T cells (Figure 4E). This niche exhibited high expression of genes involved in collagen production, such as *COL3A1* and *COL1A1*, as well as *CCL21*, a chemoattractant for immune cells (Figure 4F). In contrast, the second niche, termed the nonfibrotic niche, was predominantly composed of hepatocytes and displayed high expression of genes associated with normal liver metabolic functions, including *ADH1B* and *BAAT*. Notably, the spatial distribution of the fibrotic niche aligned with periductal regions, represented as regions with enriched cholangiocytes (Supplemental Figure 3).

### Periductal fibrosis correlates with enriched and transcriptionally reprogrammed HSCs and macrophages.

To better understand how the cellular microenvironment influences periductal fibrosis, we examined the cell-type composition and gene expression within individual periductal regions within the fibrotic niche. We defined these regions by grouping cells within a 50 μm radius (equivalent to 4–5 surrounding cells) of a cholangiocyte into bins, excluding those that contained only isolated cholangiocytes. To prevent over-segmentation of a single periductal region, adjacent bins with overlapping cells were merged. This process identified 86 distinct periductal regions (Figure 4G).

To assess the overall transcriptomic profiles of these regions, we pooled cells in each region into pseudo-bulks and performed principal component analysis (PCA). This analysis revealed 2 distinct clusters, primarily driven by differences in the fraction of HSC1 cells (Figure 4H). Based on this distinction, we categorized the regions as high- and low-fibrosis. Notably, HSC1 cells were 2.84-fold enriched in high-fibrosis regions, which also exhibited increased proportions of MoMPs, LAM-like cells, and CD8^+^ T cells (minimum fold enrichment = 1.74, Figure 4I).

The enrichment of HSC1 cells in high-fibrosis periductal regions was accompanied by elevated expression of fibrosis-associated genes (FDR-adjusted *P* < 0.05, log fold-change ≥ 0.6, Figure 4J). For example, *COL1A1, COL3A1,* and *COL4A1* — key markers of fibrosis known to be upregulated in PSC (18–20) — showed significantly increased expression. Additionally, Versican *(VCAN)*, a component of the early fibroproliferative matrix that facilitates fibroblast migration and myofibroblast activation (21, 22), as well as macrophage activation via TLRs (23), was upregulated. Notably, these genes exhibited an average 8-fold increase in HSC1 cells from high-fibrosis regions compared with those in low-fibrosis regions. Given the coordinated upregulation of matrix-remodeling and immune-associated genes in HSC1, we next asked whether HSC1 programs in periductal niches were linked to the local macrophage microenvironment. At the periductal-niche level, we related HSC1 gene expression to nearby macrophage abundance and an inflammatory/LAM-like program. The HSC1 genes most strongly associated with these macrophage features included genes with putative roles in matrix remodeling (COL4A1, CCDC80) (24–26) or immune modulation (GREM1, CXCL13) (27–29). Among them, CXCL13 showed a strong positive correlation with both macrophage abundance and the inflammatory/LAM-like program (Supplemental Figure 4A). Importantly, HSC1 was the dominant source of CXCL13 in highly fibrotic niches (Supplemental Figure 4B). Together, these findings suggest that HSC1 cells in fibrotic periductal regions are associated not only with matrix-producing programs but also with a macrophage-enriched local microenvironment.

Although macrophage populations in high- and low-fibrosis periductal regions showed no significant differences in the expression of genes linked to complement activation (*C1QA/B/C*) or the NF-κB activation complex (*TNFAIP2/3, MCL1, GPR183*), we observed distinct transcriptional changes associated with macrophage function (FDR-adjusted *P* < 0.05, log fold-change ≥ 0.6). Specifically, genes involved in macrophage migration (*ITGB2, CXCR4, CD53*), activation (*IFNGR1, LYZ*), and fibrotic responses (*GPNMB, PSAP, LGMN*) were significantly upregulated in LAM-like and MoMP cells within high-fibrosis regions. Together with the association of HSC1-derived CXCL13 with local macrophages, these findings support coordinated remodeling of both HSC1 and macrophage programs in fibrotic periductal niches.

### CCC analysis predicts macrophages as a potential driver for fibrogenic HSC activation.

The close spatial association between HSC1 and macrophages, along with the upregulation of key fibrogenic genes in HSC1 within the fibrotic niche, suggests a functional link between the gene expression programs of these 2 cell types. To systematically investigate this relationship, we applied the Spacia algorithm to identify CCC between HSC1 and its neighboring cells in the fibrotic niche (30). Spacia prioritizes single-cell-level CCCs by incorporating cell-cell proximity as a constraint and selecting interactions that lead to downstream changes in signal-receiving cells. We predicted CCCs by modeling HSC1 gene expression as a function of signal-sending genes expressed in its neighboring cells, including CD4/8^+^ T cells, macrophages, and cholangiocytes. Genes selectively expressed in these neighboring cell types were considered candidate signal-sending genes, while genes upregulated in HSC1 served as response candidates. For each signal-response gene pair, we estimated 2 key parameters: the interaction score (β), which reflects the impact of the CCC on response gene expression, and the proximity coefficient (b), which indicates the dependency of CCC on cell proximity. Importantly, because these predictions are made at the gene-expression level, CCC should be interpreted as strong cell proximity–dependent associations between genes expressed in different cells, rather than as evidence of physical interactions between the corresponding protein molecules.

**Figure 5:**
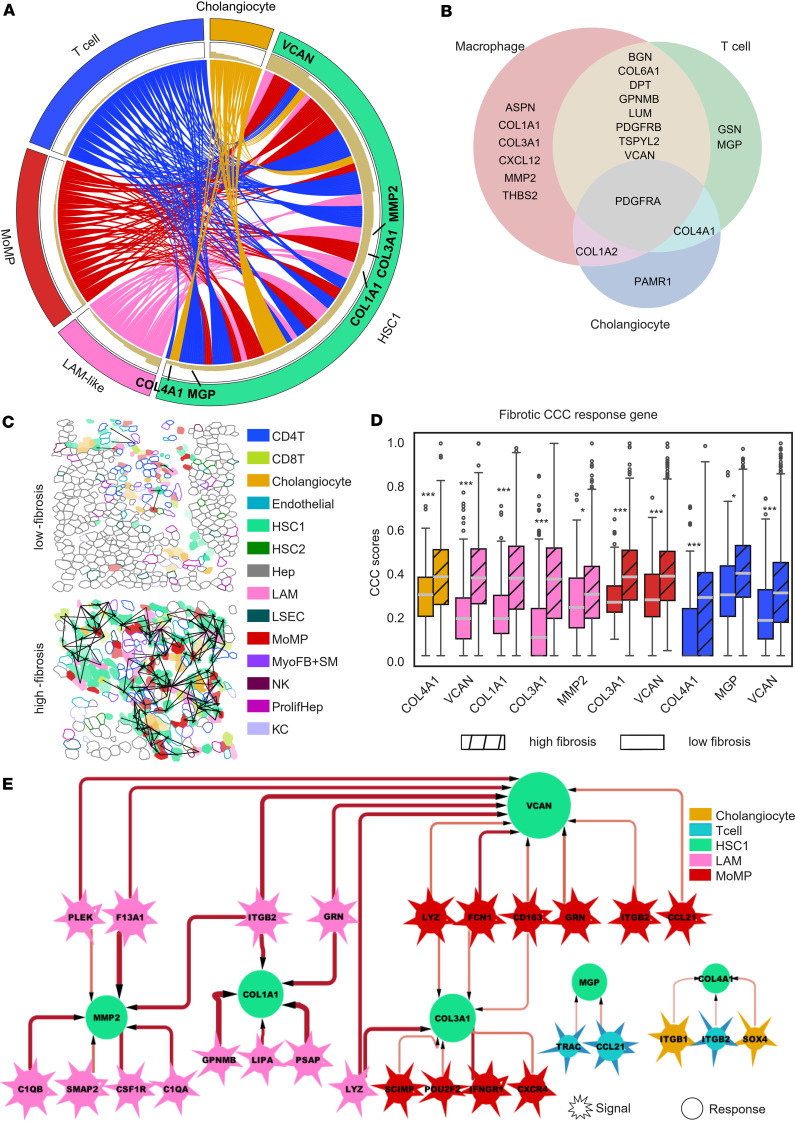
CCC analysis of periductal fibrotic niches in PSC. (**A**) Circos plot summarizing HSC1-targeting CCC from macrophages, T cells, and cholangiocytes. Each band represents a CCC between the sender cell and HSC1. The bar plot below each gene in the middle track represents the sum of absolute β values of each CCC involving this gene. CCCs were predicted between HSC1 (receiver cell) and macrophages/T cells/cholangiocytes (sender cell). (**B**) Venn diagram showing the 20 HSC1 genes targeted by sender cells. (**C**) Reconstructed images of representative low- and high-fibrosis periductal regions. Polygons in the image represent segmented cell masks and are colored based on cell types. CCCs between cells are shown as directed arrows. (**D**) Comparison of primary instance scores in HSC1 fibrogenic genes between low- and high- fibrosis periductal regions. Colors of bars represent cell types shown in **C**. *P < 0.05, ***P < 0.001. Whiskers were extended to 1.5 times IQR from Q1 and Q3. (**E**) CCC network focusing on HSC1 fibrogenic genes. Nodes with spiky borders represent genes expressed by sender cells, and nodes with smooth borders represent HSC1 genes. Nodes are colored based on cell types. CCCs are shown as directed edges, and the absolute β values are positively proportional to edge thickness.

After filtering interactions based on statistical significance (*P* values of β and b), we identified 236 HSC1-targeting CCCs originating from neighboring cells, with nearly half of these interactions driven by MoMPs or LAM-like cells (Figure 5A). Among the 20 genes in HSC1 potentially upregulated by the sender cells, several were linked to fibrosis-related processes, including collagen formation (*COL1A1, COL1A2, COL3A1, COL6A1*), PDGF signaling (*PDGFRA, PDGFRB, THBS2*), extracellular matrix remodeling (*BGN, LUM, DPT, VCAN*), and chemotaxis (*CXCL12*). Interestingly, although T cells were associated with genes involved in extracellular organization and PDGF signaling, the major collagen genes linked to liver fibrosis (23) — *COL1A1, COL1A2,* and *COL3A1* — were predominantly associated with macrophage-derived signals (Figure 5B).

To assess the spatial distribution of these CCCs, we predicted the interaction probability between each macrophage, T cell, and cholangiocyte with HSC1, as estimated by the primary instance score in Spacia. As expected, these CCCs were restricted to cells in close proximity. Moreover, HSC1-targeting CCCs were confined to fibrotic niches in both liver samples and were enriched in high-fibrosis periductal regions (Figure 5C), where HSC1 fibrotic genes — including COL1A1, COL3A1, COL4A1, MMP2, MGP, and VCAN — were upregulated. To further quantify the intensity of CCCs in high- versus low-fibrosis periductal regions, we compared the primary instance scores for each interaction between HSC1 and its neighboring cells. CCCs in high-fibrosis regions exhibited stronger interaction scores across all sender cell types, with macrophage-derived interactions targeting COL1A1, COL3A1, and VCAN displaying the most pronounced differences (Figure 5D). A network representation of these interactions revealed that HSC1 fibrotic gene upregulation was predominantly associated with macrophages, with additional contributions from T cells and cholangiocytes (Figure 5E). Specifically, COL1A1 was primarily associated with LAM-like cells, and its expression in HSC1 cells was positively correlated with genes involved in macrophage activation (GPNMB), TLR signaling (ITGB2), and fibroblast crosstalk (GRN) (31). Meanwhile, COL3A1 was mostly associated with MoMPs and was positively associated with genes linked to inflammatory responses (IFNGR1, POU2F2) and cell recruitment (CXCR4). In summary, macrophage-HSC1 crosstalk is both enriched and intensified in high-fibrosis periductal regions and associated with upregulation of HSC1-derived fibrogenic genes.

### Orthogonal validation of fibrotic macrophages and HSC signatures.

**Figure 6:**
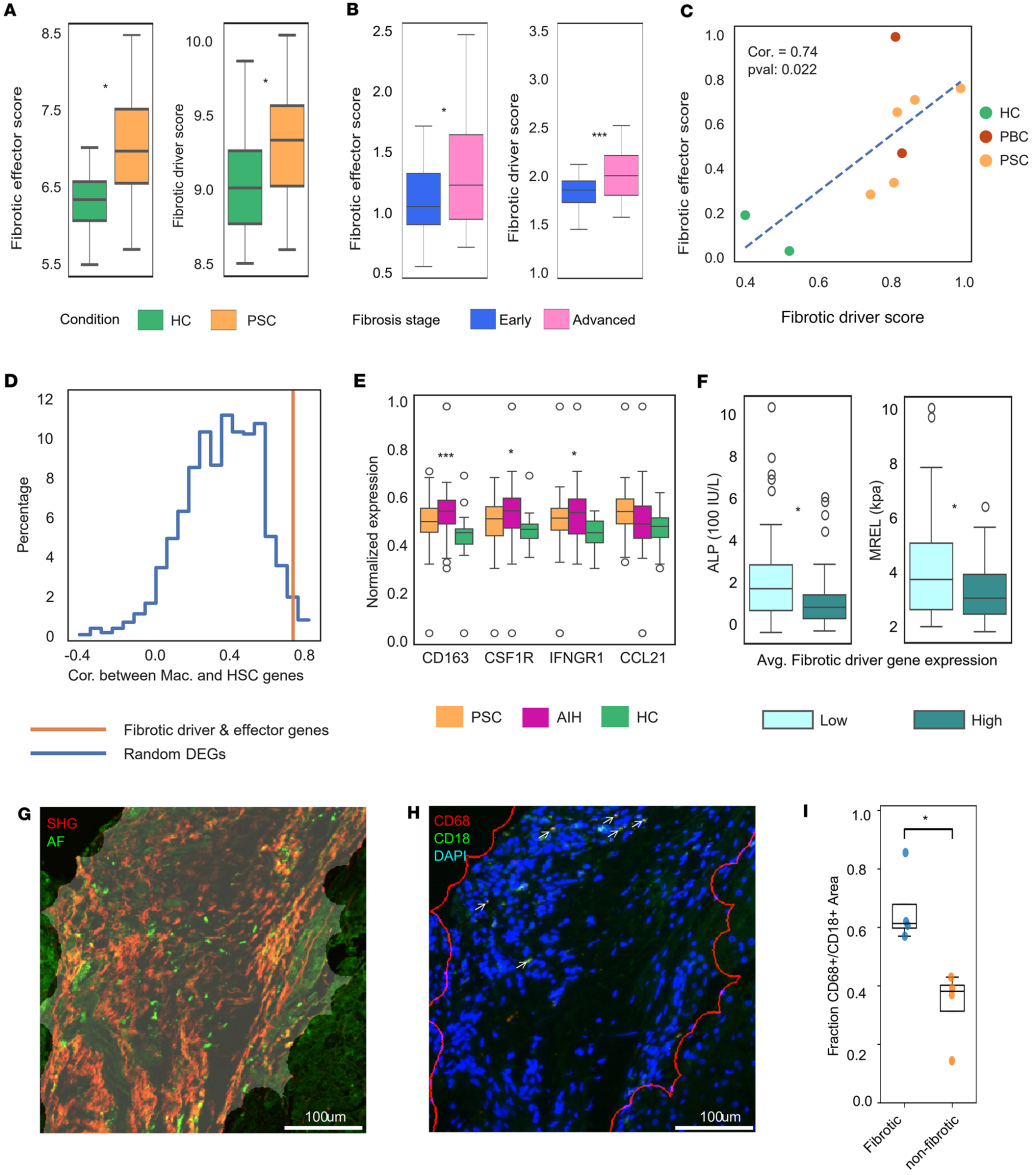
Orthogonal validation of effector and driver fibrotic signatures. (**A**) In silico validation of fibrotic driver and effector score in bulk liver RNA-Seq data from healthy controls (HCs) and adult patients with PSC. (**B**) Fibrotic driver and effector score in pediatric patients with PSC with early or advanced stages of fibrosis, which is defined as METAVIR ≥ 2 or Ishak ≥ 3. (**C**) Pearson’s correlation between fibrotic driver scores calculated in macrophages and fibrotic effector scores calculated in fibroblasts. (**D**) Comparison between baseline correlation between macrophage and fibroblast DEGs, and correlation between the fibrotic driver and effector scores. Blue line represents the distribution of the baseline correlation in 1,000 simulations using randomly sampled DEGs. Orange line represents the observed correlation between the fibrotic driver and effector scores, calculated in macrophages and fibroblasts, respectively. (**E**) Boxplot showing proteins that are elevated in PSC/AIH compared with HC in plasma. Statistical test was done using 1-way ANOVA. (**F**) Bile duct injury and liver stiffness measures in patients with AILD with high- or low- average plasma concentrations for fibrotic driver markers. Whiskers were extended to 1.5 times IQR from Q1 and Q3. (**G**) SHG image of a representative fibrotic area. Fibrotic regions were defined as SHG-positive and are highlighted. Scale bar: 100 μm. (**H**) Immunofluorescence image showing the distribution of CD68^+^/CD18^+^ macrophages. Double-positive macrophages are indicated by white arrows, and fibrotic regions are outlined in red. Scale bar: 100 μm. (**I**) Quantification of CD68^+^/CD18^+^ area in fibrotic and nonfibrotic regions. Statistical significance was determined by Student’s *t* test. *P < 0.05, ***P < 0.001.

To validate the role of macrophages in driving fibrogenic gene expression in HSC1 cells, we analyzed both bulk and scRNA-Seq data from patients with AILD, assessing the correlation between macrophage and HSC1 fibrotic gene programs and their association with PSC. Based on the predicted CCCs, we extracted 2 gene signatures from our SRT data: one representing HSC1-derived fibrotic effector genes (COL1A1, COL3A1, COL4A1, MGP, MMP2, and VCAN) and another for macrophage-derived fibrotic driver genes (Supplemental Table 3). To assess the activity of these gene signatures, we defined the fibrotic effector score as the average expression of the fibrotic effector genes, and the fibrotic driver score as the weighted average expression of the fibrotic driver genes, using Spacia-derived β coefficients as weights. We first calculated these scores in a public bulk RNA-Seq dataset from adult patients with AILD (32). In this dataset, both the fibrotic driver and effector scores were significantly higher in patients with PSC compared with healthy controls (HCs) (driver score *P* = 0.014; effector score *P* = 0.033, Figure 6A). Similarly, in our in-house RNA-Seq data from pediatric patients with AILD (30 AIH, 34 PSC), both scores were elevated in patients with advanced fibrosis (driver score *P* = 0.031; effector score *P* < 0.001, Figure 6B).

Next, we leveraged a public scRNA-Seq dataset from healthy and PSC/PBC donors (10) to examine the correlation between the fibrotic driver and effector gene signatures. The driver and effector scores were calculated at the single-cell level in macrophage and fibroblast populations, respectively, and then aggregated at the patient level. We observed a strong correlation between these scores (Pearson’s correlation = 0.74, *P* = 0.022, Figure 6C). To ensure this correlation was not simply due to these genes being among the top expressed genes in macrophage and fibroblast populations, we conducted a control analysis using randomly sampled top DEGs specific to these cell types. Although macrophage- and fibroblast-specific DEG expression was moderately correlated at the patient level (average Pearson’s correlation = 0.374), the observed correlation between the fibrotic driver and effector signatures was significantly stronger than this baseline (empirical *P* = 0.021, Figure 6D). These findings suggest that the upregulation of macrophage fibrotic driver genes is specifically associated with increased expression of fibrotic genes in fibroblasts within the same patient.

To determine whether the macrophage fibrotic gene signature is also reflected at the protein level in plasma, we performed SomaScan-based proteomic profiling using plasma samples from HCs (*n* = 22), pediatric patients with AIH (*n* = 43), and patients with PSC (*n* = 43; Table 3). Eight out of 17 macrophage fibrotic drivers (LYZ, GRN, GPNMB, FCN1, CD163, CSF1R, IFNGR1, and CCL21) were included in the SomaScan panel. Among them, plasma concentrations for CD163, CSF1R, IFNGR1, and CCL21 were increased in patients with AILD compared with HCs (Figure 6E). Next, we calculated the fibrotic driver score based on the average expression of available proteins in the SomaScan panel. Patients were dichotomized based on this score, with those above the median assigned to the high group and those below to the low group. Clinical measures were compared between the 2 groups. Markers of hepatocellular injury (i.e., serum aspartate aminotransferase [AST] and alanine aminotransferase [ALT]) did not differ between the groups (data not shown). However, ALP levels — a biomarker of bile duct epithelial injury — were elevated in patients with higher fibrotic driver scores (ALP *P* = 0.025; Figure 6F). Out of the 108 plasma samples, 63.5% were collected with a median of 0.35 months before or after a research MR elastography examination. We had shown before that MR elastography–derived liver stiffness values predicted histological stage of fibrosis in pediatric AILD (33). In this study, patients with higher fibrotic driver scores exhibited significantly higher liver stiffness values by MR elastography (4.2 kPa vs. 3.1 kPa, *P* = 0.014, Figure 6F).

To further validate the putative role of macrophages as a local fibrotic driver predicted by our SRT analysis, we performed immunofluorescence and second harmonic generation (SHG) imaging on liver biopsies from 4 pediatric patients with PSC with established fibrosis, matching the samples used for scSRT. SHG was used to define collagen-rich fibrotic regions (Figure 6G), and immunofluorescence was used to identify CD68^+^/CD18^+^ macrophages (Figure 6H), with CD18 corresponding to ITGB2, one of the top macrophage fibrotic driver genes linked to HSC1 in our CCC analysis. Comparing the CD68^+^/CD18^+^ area between fibrotic and nonfibrotic regions within the same sections showed that CD68^+^/CD18^+^ macrophages were significantly enriched in fibrotic regions (66.3% vs. 33.4%, *P* = 0.012, Figure 6I). These data provide protein-level validation of the macrophage enrichment pattern identified by SRT in fibrotic PSC regions.

## Discussion

Macrophages have been implicated as key drivers of liver fibrosis in adult end-stage liver disease. Building on these findings, we investigated macrophage-fibroblast crosstalk in pediatric-onset PSC across the disease severity spectrum. To this end, we applied an integrative multiomics approach — combining scSRT, bulk RNA-Seq, and SomaScan plasma proteomics — to liver biopsies and plasma samples from children and adolescents with AILD. Using scSRT, we identified fibrotic periductal niches enriched in MoMPs and LAM-like but not Kupffer cells, alongside a population of highly activated HSCs expressing high levels of fibrogenic genes. These niches also included cholangiocytes, T cells, and B cells, recapitulating classical PSC histopathology such as concentric fibrosis and ductular reaction. To account for spatial heterogeneity and to discern the cellular crosstalk between these cell types, we segmented periductal regions and applied Spacia to model CCC, predicting that macrophages expressing *LYZ, GRN, GPNMB, FCN1, CD163, CSF1R, IFNGR1*, and *CCL21* induce a profibrogenic transcriptional program in HSCs, characterized by upregulation of *COL1A1*, *COL3A1, COL4A1, MGP, MMP2,* and *VCAN*. These macrophage-fibroblast interactions were validated across independent datasets, including adult PSC single-cell and bulk RNA-Seq, and were strongly associated with advanced fibrosis in a pediatric AILD cohort (*n* = 64). Consistent with these transcriptomic findings, immunofluorescence and SHG imaging showed enrichment of CD68^+^/CD18^+^ macrophages in fibrotic regions of PSC liver biopsies, supporting the spatial pattern predicted by SRT. Furthermore, a plasma cytokine signature derived from these macrophage genes *(CD163, CSF1R, IFNGR1, CCL21*) correlated with elevated liver stiffness in 102 pediatric patients with AILD, supporting their utility as noninvasive biomarkers. Collectively, our findings define a conserved profibrotic macrophage-fibroblast crosstalk in PSC and establish a robust framework for spatially resolved CCC analysis in fibro-inflammatory liver diseases.

MoMPs have been linked to hepatocyte loss and ductal reaction (34). Notably, macrophage populations enriched around cholangiocytes in PSC livers have been reported to exhibit high C1QA/B, moderate CD163, and low-to-moderate TREM1 and TNF gene expression (35), aligning with the MoMP phenotype observed in the periductal regions of our scSRT data. This gene expression profile of the MoMP population suggests it has reduced inflammatory potential, which has been reported in vitro, where PSC macrophages showed reduced capacity to produce TNF upon activation (10). A LAM-like population has been reported in PSC and cirrhosis (10, 11, 36), with high expression of genes including *CD9, TREM2, GPNMB,* and *LGMN*. Consistent with our findings, these macrophages were shown to be enriched in periductal fibrotic regions in PSC livers (10). Given the enrichment of MoMPs and LAM-like cells in HSC-rich periductal areas, we modeled intercellular crosstalk between these cell types. Although spatial heterogeneity in gene expression often introduces analytical uncertainty, we leveraged this variability by focusing on HSC genes specifically upregulated in regions with elevated fibrosis. The cell-specific gene programs underlying this crosstalk were initially derived from a limited dataset (*n* = 2). To address this limitation and enhance the robustness of our predictions, we validated these programs using 2 independent and larger patient cohorts, and further prioritized genes involved in macrophage-HSC interactions. Among the prioritized genes, CD163 is a well-established biomarker of poor prognosis in PSC (37, 38). CCL21 is upregulated in PSC and plays a role in the development of secondary lymphoid structures and chronic inflammation. It acts on CCR7, which is expressed in cultured HSCs, promoting their activation in vitro (39); however, its profibrotic role in vivo has not been previously reported. Additionally, CSF1R has been proposed as a therapeutic target for inflammatory diseases (40), although its plasma levels have not been linked to liver fibrosis in PSC before.

scSRT is an emerging, promising tool to study pathogenesis mechanisms in disease, as it is robust against cell-type biases imposed by the cell preparation steps that are common in single-cell RNA-Seq and allows highly desired analysis, such as cell niche profiling and cell proximity–constrained CCC predictions. However, several data quality issues could limit this promise. The first limitation in using scSRT for CCC prediction is rooted in its dependency on cell segmentation. Errors in this process could lead to erroneous identification of coexpression within the same cell as CCC. In this study, we mitigated this issue by applying the Baysor segmentation algorithm and limiting the CCC search space to genes specifically expressed in sender/receiver cell types. Secondly, scSRT data are highly sparse and thus highly susceptible to the dropout effect typical in single-cell expression data. This issue can be mitigated by applying gene expression denoising or dropout correction tools to the scSRT data, such as Sprod, which we used in this study to correct gene expression noise (12).

Our results were derived and validated primarily using data from pediatric patients with AILD, where co-occurrence of AIH in PSC is more common compared with adults. Additionally, the heterogeneity of PSC — such as the distinction between large-duct and small-duct PSC — poses challenges for interpretation. These factors may limit the generalizability of our findings in adult patients with PSC. Like most studies involving scSRT, our discovery potential is limited to the predefined gene panels. However, with the emergence of whole transcriptome scSRT platforms such as CosMX WTX, future studies may expand our CCC findings by modeling the direct effectors such as cytokines, chemokines, and growth factors, and evaluate their impact on bile duct injury. Our results demonstrate the power of high-resolution scSRT to uncover pathogenic mechanisms through CCC modeling. We present a comprehensive analytical framework that addresses scSRT data quality challenges and incorporates cell niche analysis, refined segmentation, and robust CCC prediction. This framework offers a powerful and flexible strategy for dissecting cellular interactions in fibrotic disease and for advancing future spatial omics research.

## Methods

### Sex as a biological variable

Both male and female participants were included in this study, and sex was recorded for all samples. The study was not designed or powered to detect sex-specific differences, and no analyses by sex were performed. Sex distribution for all the datasets generated in this study is included in the Supplemental tables.

### Study approval and study cohort information

Patients with pediatric onset AILD receiving care at Cincinnati Children’s Hospital Medical Center were enrolled in the CALD observational study (registered as ClinicalTrials.gov NCT03175471) between February 2017 and July 2023. In this IRB-approved study (IRB 2016-7388), blood was collected from patients with an established or suspected diagnosis of AIH or PSC. The clinical diagnosis of PSC was assigned based on established guidelines (2). Patients were assigned the diagnosis of AIH if they met the International Autoimmune Hepatitis Study Group’s simplified criteria (41) and did not have radiological or histological evidence of cholangiopathy. Research MRI examinations were performed on the AILD cohort (NCT03175471, IRB 2016-7388), as previously described (42). Briefly, MRI was performed at a field strength of 1.5 T (Ingenia; Philips Healthcare, Best). Axial 2D spin-echo echo-planar MR elastography was performed through the mid-liver at 4 levels and analyzed as previously described (42). Clinical pathology reports from diagnostic liver biopsies were reviewed and assigned a fibrosis stage. Among these cases, H&E- and trichrome-stained liver sections from 41 patients were available and were independently reviewed by a pathologist, who was blinded to clinical information, to confirm AILD diagnosis and fibrosis stage.

### Immunofluorescence and SHG imaging

Unstained slides from archived, clinical liver biopsies of 4 patients with PSC were subjected to both SHG to detect type I and III collagen (43) and immunofluorescence for macrophage markers. First, the slides were preheated at 55°C overnight in an incubator, then de-paraffinized using Histoclear washes (3 times), followed by washes with 100%, 95%, and 70% ethanol. Ethanol was removed by keeping the slides in Milli-Q water and then cover-slipped with water. After cover-slipping, SHG was performed by scanning the slides on a Nikon FN1 upright multiphoton confocal laser scanning microscope (with Nikon A1plus camera). A single photon laser and resonant scanner were used to capture the whole slide images with 488 nm (GFP) channel at 4× objective applying NIS Elements software (5.42.06, build 1821, LO, 64 bit, Nikon). For SHG/2-photon emission fluorescence (TPEF) imaging, the objective was manually switched to 20× (Plan Apo VC 20× DIC N2), and Multiphoton Coherent Chameleon II TiSapphire IR laser (ranging from 700 to 1,040 nm) settings were used. Only 2 laser channels were used for imaging: autofluorescence (GFP 840 nm excitation; green) and SHG (840 nm excitation; red). The slides were scanned using the XY motorized stage by setting custom multi-points with Galvano scanner and *Z*-stacks. The images were saved in both Tiff and ND2 format.

After the slides were subjected to SHG and TPEF microscopy, the slides were transferred back into a container with Milli-Q water, and the coverslips were carefully removed. The slides were then washed in 1× TBST for 5 minutes followed by antigen retrieval with 1× AR6 buffer (Akoya Biosciences) for 15 minutes in a pressure cooker and subsequent cooled down to room temperature (RT) for 1 hour. After washing the tissues twice with 1× TBST, the slides were blocked for 1hour with 5% normal goat serum in a dark incubation box. The slides were then incubated with a cocktail of primary antibodies that contained anti-CD68 (1:300; Abcam, ab955) and anti-CD18 (1:50; Abcam, ab307406) at 4°C overnight. After 3 washes for 10 minutes each in TBST, a cocktail of secondary antibodies with goat anti-mouse AF647 (Abcam, ab150115) and goat anti-rabbit AF 594 (Invitrogen, A11012) was applied for 1 hour at RT for detection of CD68 and CD18, respectively. DAPI was applied for 10 minutes after the washes. Last, the slides were cover-slipped with Prolong Diamond Antifade Mountant without DAPI (Thermo Fisher Scientific). After the mounting media dried, the slides were imaged and scanned with a widefield Nikon TiE inverted SpectraX microscope using 20× objective and NIS Elements software (5.42.06, Nikon), and images were overlayed with images from SHG/TPEF microscopy of the same slides.

### SomaScan plasma proteomics profiling

For the SomaScan (1.3 k protein assay) proteomic study, 108 plasma samples from 43 AIH, 43 PSC, and 22 HC donors were used. Data collection, protein quantification, and quality control steps were performed at the Genome Technology Access Center in the Department of Genetics at Washington University School of Medicine, as previously described (44). SomaScan data were normalized using log and quantile transformation. The macrophage fibrotic driver score was calculated by averaging the normalized expression of the involved proteins. SomaScan samples were then divided into 2 groups based on the median of the fibrotic driver scores. Mean testing of clinical measures was done using Student’s 2-tailed *t* test.

### RNA-Seq data generation and analysis

#### RNA-Seq data acquisition.

Excess liver tissue from clinically indicated liver biopsies from 34 PSC and 30 AIH patients were used for bulk liver RNA-Seq under IRB 2017-2284 and IRB 2016-7388. Liver tissue samples were stored in RNALater ((https://www.thermofisher.com/us/en/home/brands/product-brand/rnalater.html) in –80°C. RNA was isolated with the miRNeasy Mini kit (QIAGEN) according to the manufacturer’s instructions. RNA integrity was confirmed by Agilent assays. RNA-Seq was performed by the University of Cincinnati DNA core using 101 bp paired-end reads at a read depth of 50 million bp, as reported before (45).

The alignment of reads to the human reference genome (GRCh38) was done using STAR (46) (v2.7.2b). Transcript quantification was done using FeatureCounts (40) (v1.6.4). The R package DEseq2 (47) (v1.26) was used to normalize gene counts and to generate variance stabilized count matrix. The Python package scanpy (48) (v1.9) was used for unsupervised clustering and differential expression analysis. Cell types were annotated by comparing cluster-specific DEGs with scRNA-Seq–derived cell-type signatures from human PSC samples (10). Cutoff values of absolute fold-change greater than 1.5 and FDR less than 0.05 were used to select for DEGs between sample-group comparisons. GSEA was performed using the Enrichr API (49) with the Reactome_Pathways_2024 reference library. Sets of genes representing macrophage subsets and activities (9) were added to this library. The reference scRNA-Seq dataset was download from CELLxGENE (https://cellxgene.cziscience.com/collections/0c8a364b-97b5-4cc8-a593-23c38c6f0ac5). UMAP embeddings were recalculated using the 400 MERSCOPE genes.

### VizGen MERSCOPE SRT data generation

#### Sample preparation.

Needle biopsies from 2 patients with PSC with early-stage disease were collected under IRB protocols 2017-2284 and 2016-7388. Freshly cut, unstained sections from archived FFPE liver biopsies of these 2 patients were mounted to slides provided by VizGen for hybridization according to the vendor’s instructions.

### SRT data preprocessing, normalization, and quality control

#### Raw image data preprocessing.

Spatial gene expression was decoded using the MERlin pipeline (50) on the VizGen MERSCOPE platform (software version 234b). Image stacks from multiple MERFISH rounds were aligned, background noise was filtered, and RNA spot detection was enhanced. Individual RNA molecule barcodes were decoded using a pixel-based algorithm with an adaptive barcoding scheme that corrected misidentified barcodes not matching the provided codebook. Raw image tiles of DAPI (nuclear staining), poly-T (cytoplasmic and nuclear staining), and cell boundaries (cell membrane IHC) were compiled into mosaic images across 7 z-planes using MERSCOPE software.

#### Cell segmentation.

Cell segmentation was performed using a VizGen-optimized implementation of the Cellpose algorithm (51) on the DAPI and cell boundary images. For each segmented cell, a unique identifier, outline mask, x-y-z coordinates, and shape metrics were recorded. Transcript molecules were assigned to cells based on spatial overlap with the cell masks. The total number of transcripts per gene per cell was then aggregated into a cell-by-gene expression matrix for downstream analysis.

#### Preprocessing and quality control.

Low-quality cells identified as those with fewer than 20 transcripts were removed from further analysis. Library size normalization, unsupervised clustering, and differential expression analysis were performed using the Python scanpy package (48) (v1.9, 29409532). Baysor (13) (v0.7.1) was used to refine transcript-to-cell assignments. Sprod was applied to log-transformed, library size–normalized data to reduce noise in scSRT data. Cell-type annotation was performed using a reference PSC scRNA-Seq dataset based on marker gene expressions (10).

### Neighborhood analysis, cell niche discovery, and CCC prediction

#### Neighborhood enrichment analysis.

For each reference cell type m, the enrichment score of neighboring cell type n was calculated as the ratio of its observed frequency among neighbors of m cells to its expected frequency based on overall tissue abundance. The expected fraction of neighboring cell types is estimated assuming cell types are uniformly distributed across the whole slide. Enrichment scores greater than 1 indicated enrichment, and values less than 1 indicated exclusion. The significance of such enrichment was calculated using Fisher’s exact test. In this study, a cell’s neighboring cells were defined as those within 50 μm.

#### Cell niche discovery.

In this study, a cell’s niche was defined as the collective gene expression profile of its surrounding neighborhood. For each cell, the niche was computed by averaging the PCA embeddings of all neighboring cells. This resulted in a cell-by-niche embedding matrix, which was subsequently clustered using the Leiden algorithm to identify groups of cells with similar composition and expression patterns in neighboring cells.

#### Periductal region segmentation.

In this study, periductal regions were referred to as the cell neighborhood within 50 μm of clustered cholangiocytes, which represent bile ducts or ductal reaction. To avoid over-segmentation, cholangiocyte neighborhoods sharing cells were merged into 1 periductal region. Characterization of each periductal region was done by evaluating the average gene expression of all cells in the region. These analyses were done with custom scripts using the following Python packages: scikit-learn, scipy, and shapely.

#### CCC prediction.

CCC was inferred using the Spacia algorithm, which models the association between the expression of a signal gene in sender cells and a response gene in receiver cells. For each receiver cell type of interest, spatial neighborhoods containing at least 2 sender cells of a specified type were identified, forming multiple multi-cell instances (referred to as bags). Each bag included 1 receiver cell and its surrounding sender cells. A Bayesian multi-instance learning framework was then applied to estimate the probability and magnitude of CCC between the candidate signaling and response gene pairs within each bag. The receiver cell type was set to “HSC1,” and the sender cell type included CD4/8^+^ T cells, MoMPs, LAM-like cells, and cholangiocytes. Candidate response and signal genes were selected as genes differentially expressed in HSC1 and expressed in at least 20% of the cells. The radius of the cell neighborhood was set to 50 μm.

### Orthogonal validation

Four datasets were used in the in silico validation, including a public RNA-Seq dataset from adult patients with PSC and HC donors, an in-house RNA-Seq dataset from pediatric patients with AILD, an scRNA-Seq/snRNA-Seq dataset from patients with PSC, and an in-house SomaScan plasma proteomics dataset from pediatric AILD and HC donors. Normalized expression matrix of the adult RNA-Seq dataset (52) was downloaded from the NCBI’s Gene Expression Omnibus (GEO GSE61260). scRNA-Seq/snRNA-Seq data of adult patients with PSC were downloaded from CELLxGENE as previously mentioned. Count data were normalized using library size normalization and log transformation. In RNA-Seq and SomaScan datasets, macrophage fibrogenic driver score was calculated as the average expression of the fibrotic driver genes, including *ITGB2, LYZ, GRN, F13A1, PLEK, GPNMB, FCN1, PSAP, CD163, C1QB, CXCR4, CSF1R, C1QA, LIPA, IFNGR1, SMAP2,* and *CCL21*. HSC fibrogenic effector score was calculated similarly using the fibrotic effector genes, including COL1A1, COL3A1, COL4A1, MGP, MMP2, and VCAN. In the scRNA-Seq/snRNA-Seq dataset, the macrophage fibrotic driver score was calculated as the weighted sum of the fibrogenic driver genes in macrophage populations; β values predicted by Spacia were used as the weights (Supplemental Table 4).

### Statistics

For scRNA-Seq and scSRT data, differential expression analysis was performed using Wilcoxon’s rank-sum test, with fold-change cutoff at 1.5 and *P* value cutoff at 0.05. A 2-sided 2-tailed *t* test was used to evaluate the statistical significance of macrophage signature expression between sample groups. All box plots were made with whiskers extended to data points within 1.5 times the IQR from the first (Q1) and third (Q3) quartiles of the data. Outliers were plotted as fliers.

### Data availability

The processed scSRT data including cell-by-gene counts and cell and transcript metadata are available at GEO GSE325861. Raw images associated with the study are available upon request. RNA-Seq data from 64 patients with AILD are available at GEO GSE303271. Data used to produce figures in this study are available in the Supporting Data Values file.

## Author contributions

YW, ERM, and AGM conceived of the project, obtained the funding, performed the experiments, and wrote the manuscript. YW, DA, XX, XW, GL, and ZFY analyzed the RNA-Seq, scRNA-Seq/snRNA-Seq, and scSRT data. CCR and MA recruited the study participants. MS, AM, RK, AYVH, LP, MA, and CCR generated the RNA-Seq and SomaScan data. PS performed pathological evaluations of patients involved in this study. JRD and ATT contributed to studying pathology images and reviewed the final manuscript.

## Conflict of interest

JRD has received unrelated in-kind research support from Perspectum, Philips Healthcare, GE HealthCare, Motilent, Guerbet, and Bracco Imaging and is currently serving on the Pediatric Radiology Editorial Board for Uroradiology. AGM received a research grant from Mirum Pharmaceuticals and has a consulting agreement with this company. He received travel support and lecture fees from Ipsen Pharmaceuticals. He received research support from Perspectum. ATT has consulted for GE HealthCare and has received research support from GE HealthCare, Siemens Healthineers, and Perspectum. No support from the above sources was received for the current work.

## Funding support

The content is solely the responsibility of the authors and does not necessarily represent the official views of the NIH. This work is the result of NIH funding, in whole or in part, and is subject to the NIH Public Access Policy. Through acceptance of this federal funding, the NIH has been given a right to make the work publicly available in PubMed Central.

- NIH R01DK095001 (to YW, ERM, and AGM), U01DK62497 (to AGM), and UM1TR006070 (to ERM).
- NIH P30 DK078392 (Bio-Imaging and Analysis Core, Single Cell Genomics Core, Integrated Pathology Research Core) of the Digestive Diseases Research Core Center and CALD at Cincinnati Children’s Hospital Medical Center.
- Cincinnati Children’s Research Foundation through the 2024 Center for Pediatric Genomics Award (to YW).

## Supplementary Material

Supplemental data

Supporting data values

## Figures and Tables

**Table 1:**
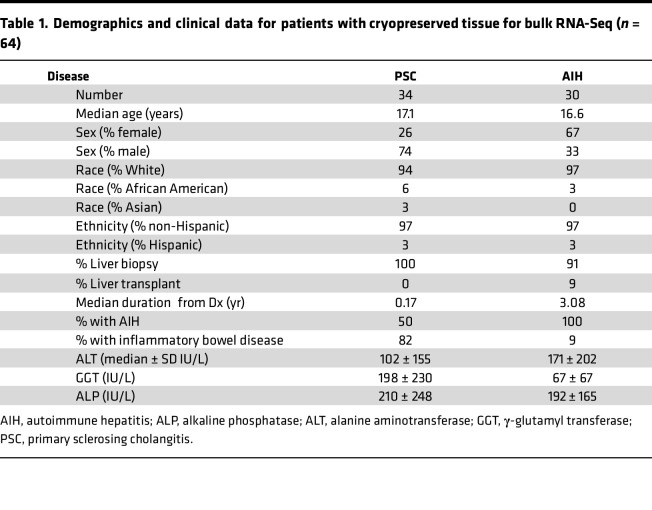
Demographics and clinical data for patients with cryopreserved tissue for bulk RNA-Seq (*n* = 64)

**Table 2:**
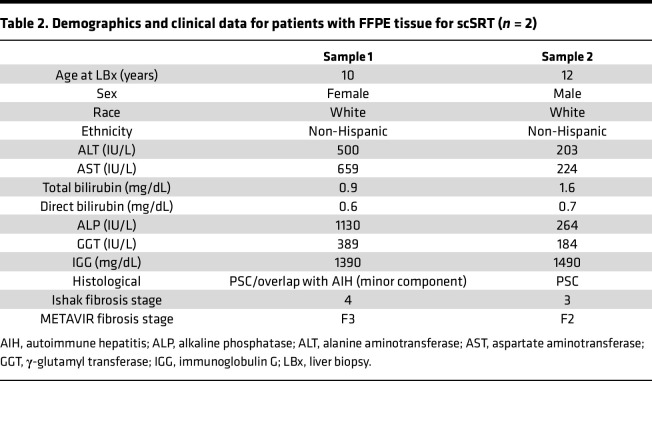
Demographics and clinical data for patients with FFPE tissue for scSRT (*n* = 2)

**Table 3:**
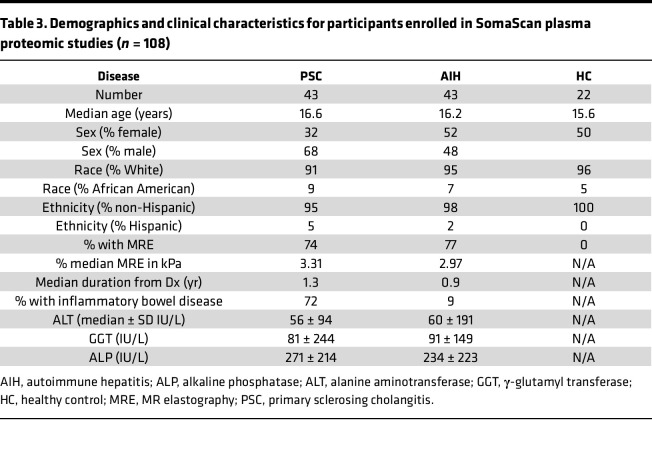
Demographics and clinical characteristics for participants enrolled in SomaScan plasma proteomic studies (*n* = 108)

## Version 1 04/23/2026

In-Press Preview

## Version 2 07/08/2026

Electronic publication

## Version 3 07/30/2026

Figure sizing error corrected
