# Supplementary material for: Macrophage signaling associates with fibrogenic program activation in periductal fibroblasts in pediatric primary sclerosing cholangitis
Source: JCI Insight. 2026 Apr 23;11(13):e199226. doi: 10.1172/jci.insight.199226 (PMC13460812; doi:10.1172/jci.insight.199226)
Supplement: Supplementary file 1 [file jciinsight-11-199226-s265.pdf]

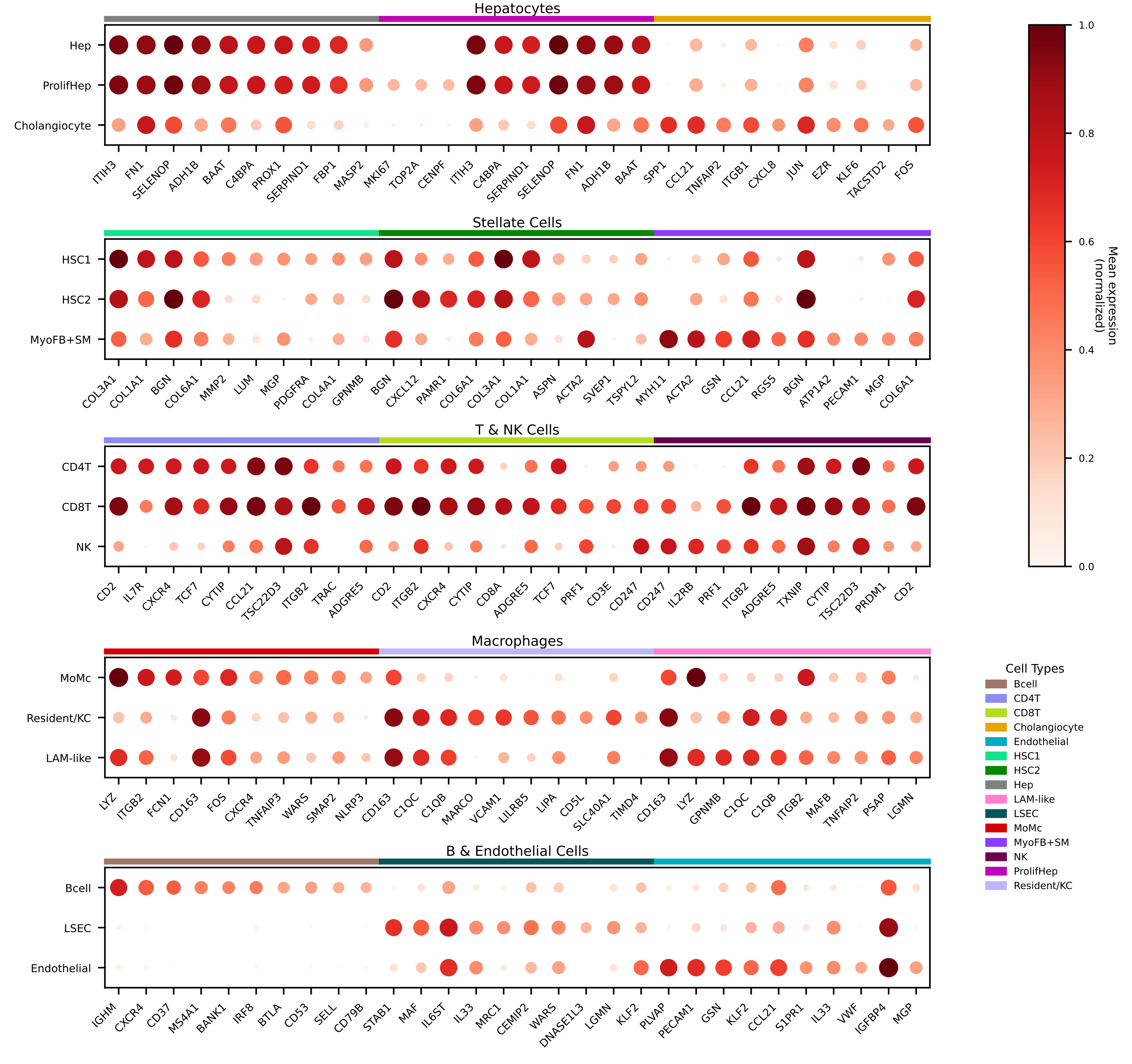
Hepatocytes
Hep
ProlifHep
Cholangiocyte
ITIH3 FN1 SELENOP ADH1B BAAT C4BPA PROX1 SERPIND1 FBP1 MASP2 MKI67 TOP2A CENPF ITIH3 C4BPA SERPIND1 SELENOP FN1 ADH1B BAAT SPP1 CCL21 TNFAIP2 ITGB1 CXCL8 JUN EZR KLF6 TACSTD2 FOS
Stellate Cells
HSC1
HSC2
MyoFB+SM
COL3A1 COL1A1 BGN COL6A1 MMP2 LUM MGP PDGFRA COL4A1 GPNMB BGN CXCL12 PAMR1 COL6A1 COL3A1 COL1A1 ASPN ACTA2 SVEP1 TSPYL2 MYH11 ACTA2 GSN CCL21 RGS5 BGN ATP1A2 PECAM1 MGP COL6A1
T & NK Cells
CD4T
CD8T
NK
CD2 IL7R CXCR4 TCF7 CYTIP CCL21 TSC22D3 ITGB2 TRAC ADGRE5 CD2 ITGB2 CXCR4 CYTIP CD8A ADGRE5 TCF7 PRF1 CD3E CD247 CD247 IL2RB PRF1 ITGB2 ADGRE5 TXNIP CYTIP TSC22D3 PRDM1 CD2
Macrophages
MoMc
Resident/KC
LAM-like
LYZ ITGB2 FCN1 CD163 FOS CXCR4 TNFAIP3 WARS SMAP2 NLRP3 CD163 C1QC C1QB MARCO VCAM1 LILRB5 LIPA CD5L SLC40A1 TIMD4 CD163 LYZ GPNMB C1QC C1QB ITGB2 MAFB TNFAIP2 PSAP LGMN
B & Endothelial Cells
Bcell
LSEC
Endothelial
IGHM CXCR4 CD37 MS4A1 BANK1 IRF8 BTLA CD53 SELL CD79B STAB1 MAF IL6ST IL33 MRC1 CEMIP2 WARS DNASE1L3 LGMN KLF2 PLVAP PECAM1 GSN KLF2 CCL21 S1PR1 IL33 VWF IGFBP4 MGP
Mean expression (normalized)
0.0
0.2
0.4
0.6
0.8
1.0
Cell Types
Bcell
CD4T
CD8T
Cholangiocyte
Endothelial
HSC1
HSC2
Hep
LAM-like
LSEC
MoMc
MyoFB+SM
NK
ProlifHep
Resident/KC

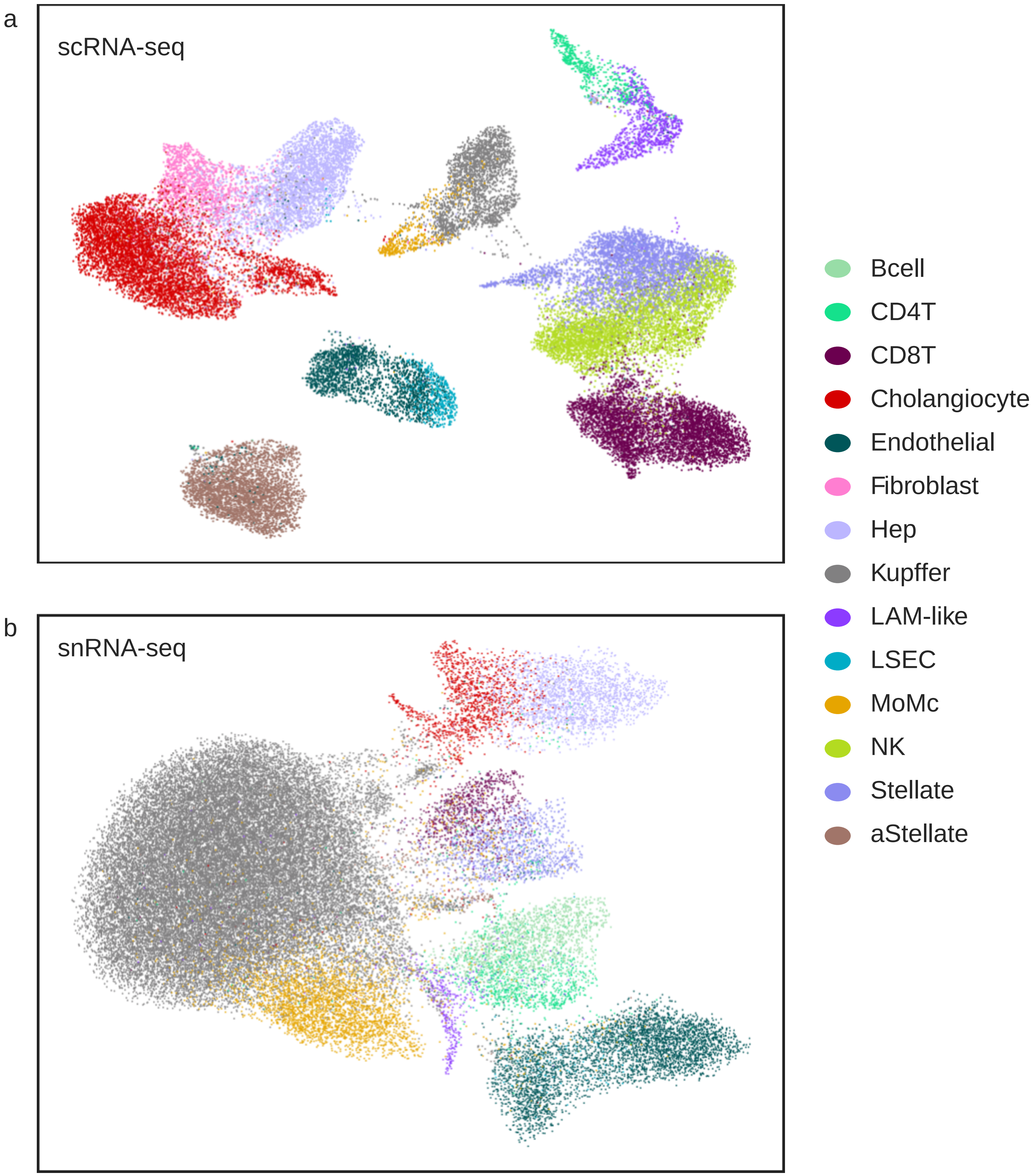

a
scRNA-seq
b
snRNA-seq
Bcell
CD4T
CD8T
Cholangiocyte
Endothelial
Fibroblast
Hep
Kupffer
LAM-like
LSEC
MoMc
NK
Stellate
aStellate

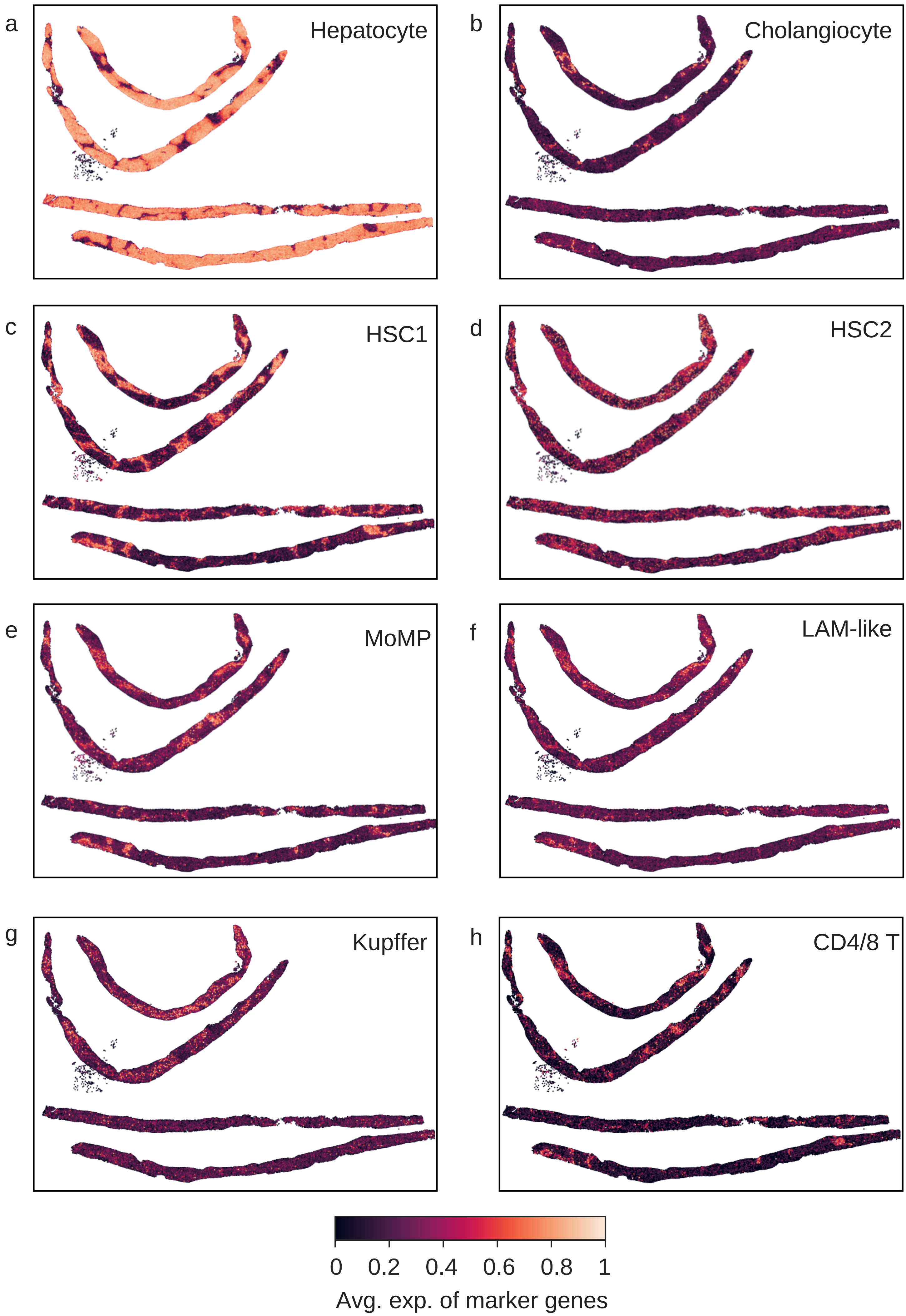
a
Hepatocyte
b
Cholangiocyte
c
HSC1
d
HSC2
e
MoMP
f
LAM-like
g
Kupffer
h
CD4/8 T
0
0.2
0.4
0.6
0.8
1
Avg. exp. of marker genes

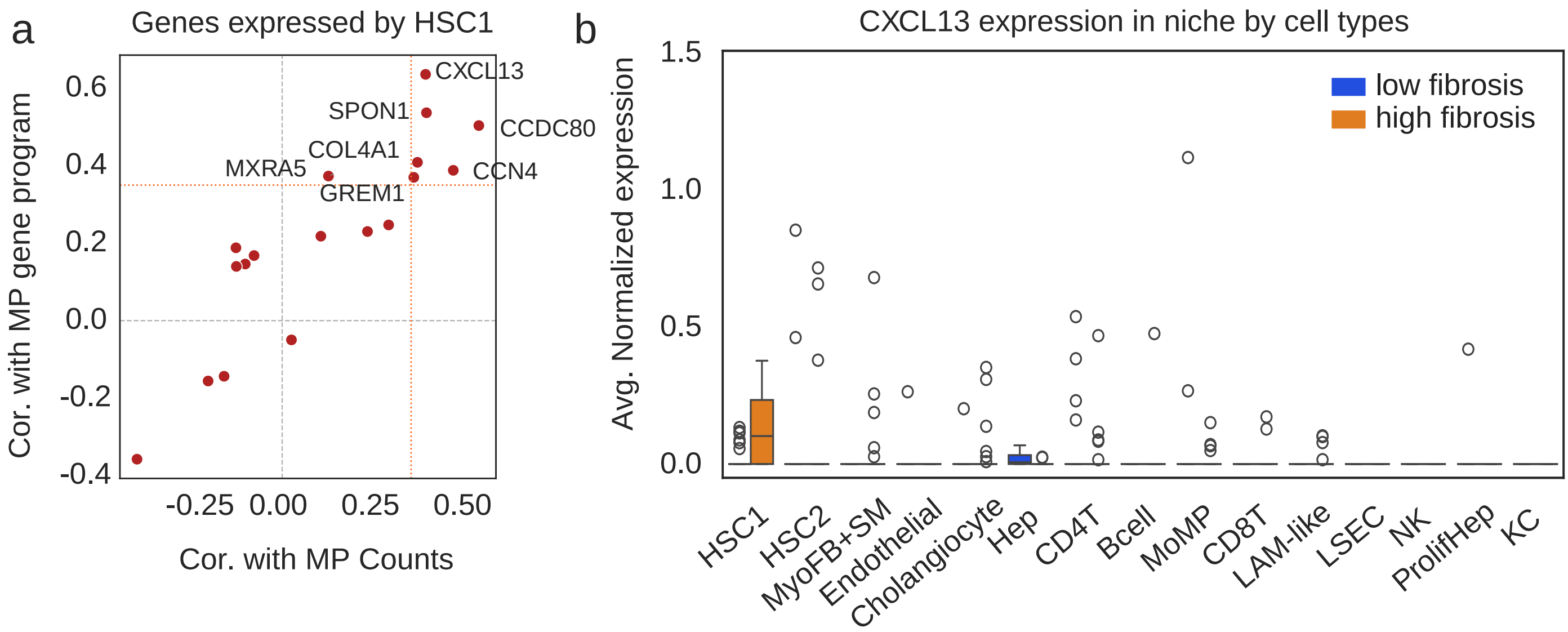
a
Genes expressed by HSC1
Cor. with MP gene program
Cor. with MP Counts
0.6
0.4
0.2
0.0
-0.2
-0.4
-0.25 0.00 0.25 0.50
CXCL13
SPON1
CCDC80
COL4A1
MXRA5
CCN4
GREM1
b
CXCL13 expression in niche by cell types
Avg. Normalized expression
1.5
1.0
0.5
0.0
low fibrosis
high fibrosis
HSC1
HSC2
MyoFB+SM
Endothelial
Cholangiocyte
Hep
CD4T
Bcell
MoMP
CD8T
LAM-like
LSEC
NK
ProlifHep
KC

Figure S1. Top differentially expressed genes in PSC cell types.
Dot plot illustrating the expression of the top 10 marker genes for each cell type. Dot size represents the proportion of cells expressing the gene, while color intensity indicates the expression level. Gene expression in log (CPM+1) was scaled to a range of 0 to 1.

Figure S2. UMAP embeddings of PSC cell types from the reference dataset.
Recalculated UMAP embeddings of cells in the reference scRNA-seq (a) and snRNA-seq (b) dataset. Clusters are color-coded based on cell types.

Figure S3: Spatial distribution of cell types identified in the scSRT PSC samples.
Panels (a–h) depict the spatial localization and average expression of cell top typespecific marker genes across FFPE liver tissue slides. Colors are mapped based on the average expression of the top 10 differentially expressed genes in each cell type. Cells are plotted based on the spatial coordinates of their centroids.

Figure S4. HSC1-derived CXCL13 is associated with local macrophage abundance and gene programs in periductal niches. (a), Spearman correlation of HSC1-derived gene expression with nearby macrophage counts and gene programs. Genes with at least modest correlation ($r >= 0.35$) are highlighted. (b), Boxplot showing CXCL13 expression across major cell types in low- and high-fibrosis niches. Whiskers were extended to 1.5 times IQR from Q1 and Q3.
